# [^18^F]Fluciclatide PET as a biomarker of response to combination therapy of pazopanib and paclitaxel in platinum-resistant/refractory ovarian cancer

**DOI:** 10.1007/s00259-019-04532-z

**Published:** 2019-11-21

**Authors:** Rohini Sharma, Pablo Oriol Valls, Marianna Inglese, Suraiya Dubash, Michelle Chen, Hani Gabra, Ana Montes, Amarnath Challapalli, Mubarik Arshad, George Tharakan, Ed Chambers, Tom Cole, Jingky P. Lozano-Kuehne, Tara D. Barwick, Eric O. Aboagye

**Affiliations:** 1 Department of Surgery and Cancer, Imperial College London, Hammersmith Campus, Du Cane Road, London, W12 0HS UK; 2 Department of Computer, Control and Management Engineering Antonio Ruberti, University of Rome “La Sapienza”, Rome, Italy; 3 Clinical Discovery Unit, Early Clinical Development, AstraZeneca, Cambridge, UK; 4 Department of Medical Oncology, Guy’s and St Thomas’ NHS Foundation Trust, London, UK; 5 Bristol Cancer Institute, UH Bristol NHS Foundation Trust, Bristol, UK; 6 Division of Diabetes, Endocrinology and Metabolism, Imperial College London, London, UK; 7 Department of Medicine, Division of Experimental Medicine, NIHR Imperial Clinical Research Facility, Imperial College London, London, UK; 8 Department of Radiology, Imperial College Healthcare NHS Trust, London, UK

**Keywords:** Pazopanib, [^18^F]Fluciclatide, Ovarian cancer, Platinum resistance, Angiogenesis, PET imaging

## Abstract

**Background:**

Angiogenesis is a driver of platinum resistance in ovarian cancer. We assessed the effect of combination pazopanib and paclitaxel followed by maintenance pazopanib in patients with platinum-resistant/refractory ovarian cancer. Integrins α_v_β_3_ and α_v_β_5_ are both upregulated in tumor-associated vasculature. [^18^F]Fluciclatide is a novel PET tracer that has high affinity for integrins α_v_β_3/5_, and was used to assess the anti-angiogenic effect of pazopanib.

**Patients and methods:**

We conducted an open-label, phase Ib study in patients with platinum-resistant/refractory ovarian cancer. Patients received 1 week of single-agent pazopanib (800 mg daily) followed by combination therapy with weekly paclitaxel (80 mg/m^2^). Following completion of 18 weeks of combination therapy, patients continued with single-agent pazopanib until disease progression. Dynamic [^18^F]fluciclatide-PET imaging was conducted at baseline and after 1 week of pazopanib. Response (RECIST 1.1), toxicities, and survival outcomes were recorded. Circulating markers of angiogenesis were assessed with therapy.

**Results:**

Fourteen patients were included in the intention-to-treat analysis. Complete and partial responses were seen in seven patients (54%). Median progression-free survival (PFS) was 10.63 months, and overall survival (OS) was 18.5 months. Baseline [^18^F]fluciclatide uptake was predictive of long PFS. Elevated baseline circulating angiopoietin and fibroblast growth factor (FGF) were predictive of greater reduction in SUV_60,mean_ following pazopanib. Kinetic modeling of PET data indicated a reduction in *K*_1_ and *K*_i_ following pazopanib indicating reduced radioligand delivery and retention.

**Conclusions:**

Combination therapy followed by maintenance pazopanib is effective and tolerable in platinum-resistant/refractory ovarian cancer. [^18^F]Fluciclatide-PET uptake parameters predict clinical outcome with pazopanib therapy indicating an anti-angiogenic response.

**Electronic supplementary material:**

The online version of this article (10.1007/s00259-019-04532-z) contains supplementary material, which is available to authorized users.

## Introduction

The negative impact of angiogenesis on survival outcomes for patients with ovarian cancer is well established [1–4]. In particular, high tumoral microvessel density (MVD) is associated with high rates of tumor recurrence and death [5–7]. Moreover, vascular endothelial growth factor (VEGF) has been shown to be upregulated in platinum-resistant ovarian cancer [8]. Consistent with the central role of angiogenesis in disease progression and survival, bevacizumab, a monoclonal antibody that targets VEGF, has been shown to be active in the management of both platinum-sensitive and platinum-resistant ovarian cancer with three large randomized controlled trials, confirming an additive benefit of anti-angiogenic therapy to chemotherapy [9–12]. However, the response rate to combinatorial therapy, particularly in the platinum-resistant setting, is modest and there is a need for more active therapeutic strategies [9].

Pazopanib is a pan-VEGF tyrosine kinase inhibitor that has additional inhibitory effects on α and β platelet-derived growth factor (PDGF) receptors, and the proto-oncogene receptor tyrosine kinase (c-KIT) [13]. Two large studies investigated the clinical utility of adding pazopanib to weekly paclitaxel, with conflicting results; with the MITO 11 study reporting a 3-month PFS benefit in patients with platinum-resistant disease and the study by Richardson and colleagues reporting no benefit, albeit in a mixed platinum-resistant and platinum-sensitive population [14, 15]. Du Bois and colleagues demonstrated an improvement in PFS following the addition of maintenance pazopanib after completion of chemotherapy in platinum-sensitive disease [16].

Despite their utility, all anti-angiogenic strategies are associated with toxicities. Importantly, the addition of pazopanib to weekly paclitaxel resulted in significant toxicities including a higher rate of grade 3–4 myelosuppression, fatigue, hypertension, and liver dysfunction [16, 17]. Given the toxicity profile and conflicting clinical outcome data, there is a real need for a validated biomarker of response to anti-angiogenic drugs in this setting such that patients are not exposed to potentially life-threatening side effects in the absence of therapeutic benefit, particularly given the palliative nature of therapy in this patient population.

Integrins are a family of cell adhesion molecules that facilitate the interaction between tumor vasculature and the extracellular matrix [18, 19]. α_v_β_3/5_-Integrins are of particular interest as these are expressed at low levels on mature vessels and non-neoplastic epithelium but are highly expressed on activated tumor-related endothelial cells [18, 20]. α_v_β_3/5_-Integrins interact with components of the extracellular matrix such as fibronectin, laminin, and collagen via the tripeptide sequence arginine-glycine-aspartic acid (RGD) [21]. Peptide ligands containing the RGD sequence have high affinity for integrin receptors, and a number of radiolabeled probes have been developed to image integrin expression with either positron emission tomography (PET) or single-photon emission tomography [22–26]. [^18^F]Fluciclatide is an [^18^F]-labeled cyclic tripeptide that contains the RGD sequence, and binds with high affinity to integrins α_v_β_3_ and α_v_β_5_, both of which are upregulated in tumor-associated vasculature, and has been shown to visualize response to anti-angiogenics in the preclinical setting [27, 28].

The aim of this study was to examine the clinical efficacy of combinatorial therapy with pazopanib and weekly paclitaxel, followed by maintenance pazopanib in patients with platinum-resistant/refractory ovarian cancer. In parallel, we also sought to ascertain whether [^18^F]fluciclatide-PET could be used as a pharmacodynamic (PD) marker of pazopanib effect following 1 week of single-agent therapy. The secondary endpoints were to assess the relationship between [^18^F]fluciclatide uptake with pharmacokinetics (PKs) of pazopanib and with circulating markers of angiogenesis.

## Materials and methods

### Study design and participants

We conducted a phase 1b study in patients with platinum-resistant and refractory ovarian cancer defined as those whose disease had progressed during first-line platinum-based chemotherapy or relapsed within 6 months after last platinum treatment. Patients were excluded if they had previously received either weekly paclitaxel or any anti-angiogenic therapy. Eligible patients were > 18 years old with cytological or histological diagnosis of epithelial ovarian, had disease evaluable by Response Evaluation Criteria in Solid Tumors (RECIST 1.1) and at least 1 target lesion > 2 cm, and had an Eastern Cooperative Oncology Group performance status (PS) of 2 or less.

Patients had to have adequate bone marrow function (hemoglobin concentration ≥ 90 g/L, neutrophils ≥ 1500 cells/μL, platelets ≥ 100,000 cells/μL), kidney function (creatinine ≤ 1.5 mg/dL or if creatinine ≤ 1.5 mg/dL a calculated creatinine clearance ≥ 50 mL/min; urine protein:creatinine ratio < 1, or for urine protein:creatinine ratio ≥ 1 the patient had to have a 24-h urine protein < 1 g), and liver function (aspartate aminotransferase (AST) and alanine aminotransferase (ALT) ≤ 2.5× the upper limit of normal (ULN), total bilirubin ≤ 1.5× ULN). Exclusion criteria included poorly controlled hypertension, defined as systolic blood pressure (SBP) of ≥ 140 mmHg or diastolic blood pressure (DBP) of ≥ 90 mmHg, and presence of clinically significant peripheral neuropathy or gross ascites. Patients were recruited from Hammersmith Hospital, Imperial College Healthcare NHS Trust and Guy’s and St Thomas’ NHS Foundation Trust, UK. The study was approved by the North of Scotland research ethics committee, ethics number 10/S0801/36. All the patients provided written informed consent. The study was conducted in compliance with the Declaration of Helsinki and registered with ClinicalTrials.gov (number NCT01608009) and EudraCT (number 2009-017993-19).

### Study assessments

All patients received 7 days of single-agent pazopanib (800 mg daily) followed by weekly paclitaxel (80 mg/m^2^) for 18 weeks. Following review of PK data, however, the paclitaxel dose was reduced to 60 mg/m^2^, such that only one patient received 80 mg/m^2^ of paclitaxel. Prophylactic supportive therapy was given according to local procedures to all patients prior to paclitaxel administration. Following completion of combination therapy, maintenance of pazopanib 800 mg daily was continued until disease progression, patient withdrawal, or unacceptable toxic effects. Pazopanib and paclitaxel dosing were modified according to toxicity as previously described [14].

Baseline staging included clinical examination; CT chest, abdomen, and pelvis; and serum CA 125 measurement. The same radiological tests were repeated after 9 weeks of combination therapy, at the end of combination treatment (18 weeks), and then 3-monthly until disease progression. Response was assessed in accordance with RECIST 1.1. CA 125 was measured every 3 weeks or earlier on clinician judgment. [^18^F]Fluciclatide PET/CT imaging was conducted at baseline, after 7 days of single-agent pazopanib and at disease progression. Limited sampling for pazopanib PKs was taken following 6 days of pazopanib therapy: prior to pazopanib dose (trough concentration, *C*_min_) and after 4 h (peak concentration, *C*_peak_).

### Outcomes

The primary endpoint was the change in [^18^F]Fluciclatide uptake parameters after 1 week of pazopanib treatment. Changes in PET uptake parameters were correlated with best treatment response (RECIST 1.1). Secondary endpoints were PFS, defined as time from commencement of pazopanib to either progression (as per RECIST 1.1) or death, frequency of adverse events (AEs) relating to pazopanib, and response according to Gynecologic Cancer InterGroup (GCIG) CA 125 response criteria [29].

### Imaging protocol

[^18^F]Fluciclatide was manufactured according to standard protocols (GE Healthcare, London) [30]. Images were acquired on a Biograph 6 TruePoint PET/CT scanner (with TrueV; extended field of view [Siemens]) with 21.6-cm axial and 60.5-cm transaxial fields of view. In all cases, target lesions were imaged in a single abdominal bed position. The field of view for the single bed position was based on the position of the largest target lesion. A non-contrast CT scan (300 mA, 120 kVp, 1.35 pitch, 0.8 s/rotation) was conducted for both attenuation correction of PET data and co-registration with PET images. [^18^F]Fluciclatide (mean (±SD) 346 + 9.9 MBq) was injected intravenously as a bolus injection and a dynamic, list mode emission scan in the 3D mode, lasting 66 min, was undertaken. Blood was collected for radioligand metabolite analysis.

### Image analysis

Raw PET data were corrected for scatter and attenuation, and reconstructed with an iterative algorithm consisting of 8 iterations and 21 subsets. The data were binned into time frames as follows: 1 × 30 s (background), 6 × 10 s, 4 × 20 s, 4 × 30 s, 5 × 120 s, 4 × 180 s, and 4 × 600 s. The attenuation-corrected PET images and CT data were fused and analyzed on a dedicated workstation (Hermes Diagnostics, Sweden) by a dual accredited radiologist-nuclear medicine physician (TB). All SUV analyses were conducted using PET uptake parameters generated on Hermes.

Tumor lesions were defined as target lesions by RECIST 1.1 on CT [31]. The lesions on the [^18^F]fluciclatide-PET imaging corresponding to those on the CT, showing an increased uptake, were considered as target lesions. The diameter of the target lesions was measured on CT using electronic calipers on the PACS workstation. All lesions greater than 20 mm on CT imaging were evaluated on PET/CT. The same target lesions were used for analyses on both the PET/CT and CT, before and after treatment.Consecutive volumes of interest (VOI) were manually defined around the tumors on the summed images, employing the patient’s diagnostic images for guidance. The VOI encompassed the whole tumor for SUV analysis. For quantification, imaging data within VOIs of individual time frames were used. The [^18^F]fluciclatide radioactivity concentrations within the VOIs were normalized to injected radioactivity and body weight (grams) to obtain the mean and maximum SUV at 60 min (SUV_60,mean_ and SUV_60,max_) on baseline and post-treatment [^18^F]fluciclatide PET/CT studies. The percentage change in both SUV_60,mean_ and SUV_60,max_ was then calculated for each target lesion visible on baseline imaging as follows: (SUV_post_ – SUV_pre_)/SUV_pre_. All target lesions were included in the final analysis.

### Quantitative analysis

Kinetic analysis was undertaken with spectral analysis (SA) and graphical analysis, with the latter exploring both Patlak and Logan plots [32–36]. A population-averaged arterial input function (AIF) was built from a previous dataset of patients who had had dynamic imaging with [^18^F]fluciclatide with arterial sampling [37]. From the previous dataset, total blood input functions were corrected for delay and averaged resulting in a population-averaged whole blood input function and used to derive the AIF for our dataset. The AIF was corrected for blood counting and metabolite analysis using the discrete blood samples obtained from individual patients during the scan at 5, 10, 15, 30, and 60 min [38].

### Circulating levels of pro-angiogenic factors

Circulating levels of placental growth factor (PIGF); endothelial tyrosine kinase receptor (TIE-2); VEGF; VEGF-D; VEGF-C; endothelin-1; endostatin; human growth factor (HGF); angiopoietin-1; VEGF receptor (VEGFR)-1, VEGFR-2, and VEGFR-3; and fibroblast growth factor (FGF) were determined at baseline and week 1. Plasma samples with lithium heparin were collected and centrifuged at 1200×*g* for 10 min at room temperature for separation of plasma and mononuclear cell layers. Plasma was stored at − 80 °C until analysis. On the day of analysis, plasma was diluted 1:2 as per manufacturer’s guidelines, and cytokines were measured using a human Luminex bead-based assay (R&D Systems, Inc. Minneapolis, MN, USA).

### Statistical analysis

Assuming a 15% response to pazopanib as detected by [^18^F]fluciclatide, the study required a sample size of 13 patients for estimating the expected proportion with 20% absolute precision and 95% confidence. Assuming a 30% dropout rate and scan failure, four patients were additionally enrolled, such that the total number of patients to be recruited was 17.

For PET analysis, CR and PR were grouped together as “responders” while SD and PD were grouped as “non-responders.” Paired student *t* test or non-parametric regression analysis was used to evaluate utility of the tracer pre- and post-pazopanib therapy depending on the distribution of the data. Association between PET parameters and expression of tumor markers and potential circulating biomarkers was investigated using the Wilcoxon rank sign correlation coefficient. ANOVA was used to compare group effects. Kaplan–Meier statistics was used for survival analyses. All analyses were two-sided, with a level of significance of ≤ 0.05. All statistical analyses were conducted using SPSS statistical package version 22 (SPSS Inc., Chicago, IL, USA).

## Results

### Patient characteristics

Between August 2012 and March 2015, 16 patients were enrolled from Hammersmith Hospital, Imperial College Healthcare NHS Trust (*N* = 14), and Guy’s and St Thomas’ NHS Foundation Trust (*n* = 2). One patient withdrew consent following the first PET scan and one patient was unable to tolerate the PET scan and withdrew; therefore, 14 patients were evaluable for primary outcome analysis. Median age was 54 years, and the mean platinum free interval (PFI) was 74 days prior to enrolment. The median duration of pazopanib treatment was 226.5 days (range 42.9–969 days). Nine (64.3%) patients had stage III disease and five (38.5%) patients had stage IV disease. In terms of previous lines of chemotherapy, four (28.6%) patients had failed first-line platinum-based therapy, two (21.4%) had had two previous lines of therapy, and seven (50.0%) had had three or more lines of systemic therapy.

### Efficacy

Thirteen patients were evaluable for treatment response (RECIST 1.1). One patient developed bowel perforation and died following 1 cycle of combination therapy so was not included in outcome analysis. One patient was unable to tolerate the second PET scan but remained in the intention-to-treat analysis. Of the RECIST 1.1 evaluable patients, one patient experienced CR (8%), six patients had a PR (46%), and stable disease was seen in five patients (38%) yielding an overall clinical benefit rate (CR + PR + SD) of 92%. One patient (8%) had progressive disease.

Twelve patients (86%) were suitable for GCIG CA125 response analysis. Of these, CA125 response was observed in 11 (92%) patients, with a mean decrease in CA125 of 92% at both weeks 9 and 18. Of the responding patients, seven patients experienced a CR to therapy based on GCIG CA125 criteria (Supplementary Fig. 1).

In terms of PFS, the median PFS was 10.6 months (95%CI 5.8–15.5) for all evaluable patients (Supplementary Fig. 2A). Univariate analysis of PFS by log rank test revealed a significant difference across response categories (*p =* 0.003). Patients experiencing CR and PR had a median PFS of 11.3 months (95%CI 0.7–21.9), SD 7.9 months (95%CI 3.9–11.9), and PD 1.6 months (95%CI not reached) (Supplementary Fig. 2B). The overall survival (OS) of the study population was 18.5 months (95%CI 6.8–30.1). Univariate analysis of OS by log rank test revealed a significant difference across response categories (*p =* 0.001). The OS in patients experiencing CR and PR was 25.3 months (95%CI 24.8–25.8), compared with 15.5 months in those experiencing SD (95%CI, 1.6–29.4) and 2.5 months in patients experiencing PD (95%CI 3.1–40.2) (Supplementary Fig. 2C).

### Safety and tolerability

All but one patient experienced an adverse event with a total number of 99 adverse events recorded, of which 49 (49.5%) were grade 1, 25 (25.2%) were grade 2, 23 (23.2%) were grade 3, and the remaining (2, 2%) were grades 4 and 5 (Table 1). Overall, diarrhea was the most frequent adverse event (all grades). The commonest grade 3 toxicity was lethargy and abnormal liver function tests (LFTs). One patient had grade 4 liver toxicity and permanently discontinued treatment. One patient died subsequent to bowel perforation that was attributed to pazopanib.

**Table 1:** Number of adverse events (CTCAE v4.03) in the entire study population related to pazopanib administration over the treatment course

Toxicity	Grade 1	Grade 2	Grade 3	Grade 4	Grade 5
Neutropenia	3		1		
Lethargy	3	7	5		
Diarrhea	12	1	1		
Mucositis	3	1	1		
Anorexia		1	1		
Nausea and vomiting	4	2	2		
Palmar plantar erythrodermatitis	2	4	3		
Abnormal LFTs			5	1	
Hypertension	1	2	1		
Other	21	7	3^		1*
Total (%)	49 (49.5)	25 (25.2)	23 (23.2)	1 (1)	1 (1)

*Bowel perforation

^Hematuria, anemia, reduced cardiac ejection fraction

### Pharmacokinetic analysis

All patients had evaluable PK samples. The mean *C*_min_ (+ SD) of pazopanib was 34 μg/mL + 13.2, while mean *C*_peak_ was 45.8 μg/mL + 14.0. Previous studies have suggested *C*_min_ of ≥ 20 mg/L as a PK threshold for efficacy. Only two patients had a *C*_min_ of < 20 mg/L, and we found no relationship between RECIST 1.1 or survival and *C*_min_ [39, 40].

### Platinum sensitivity restored

Of particular clinical significance, 9 of the 14 patients enrolled in the study (64%) were rendered “platinum sensitive” such that their disease relapsed more than 6 months after prior platinum exposure. Of these patients, five patients responded favorably to re-challenge with carboplatin-based therapy. Two patients experienced exceptional responses to maintenance pazopanib. The first patient with moderately differentiated, serous adenocarcinoma had previously received carboplatin/paclitaxel followed by carboplatin/paclitaxel/PDGF inhibitor. At trial enrolment, her PFI was 64 days. She experienced a PR to combination therapy according to RECIST 1.1 and CR according to GCIG CA125 criteria. She remained on pazopanib, having a PFS of 32 months. She was successfully re-challenged to PR with subsequent platinum therapy. The second patient with poorly differentiated, serous adenocarcinoma had previously received carboplatin/paclitaxel followed by carboplatin/caelyx. At trial enrolment, her PFI was 7 days. She experienced a PR to combination therapy but was not evaluable according to GCIG CA125 criteria. She had a PFS of 21 months.

### Circulating markers of angiogenesis

Analysis of circulating angiogenic factors prior to and following 1 week of pazopanib indicated that only PIGF significantly changed such that a 58% increase in PIGF was observed following therapy (*p* = 0.03) (Supplementary Table 1). We then considered whether levels of circulating cytokines were associated with RECIST 1.1 response. A significant relationship was observed between baseline angiopoietin-1 and RECIST 1.1 response such that elevated levels of angiopoietin-1 at baseline predicted for non-response to pazopanib (SD/PD) (*p* = 0.049, ANOVA). No relationship was observed between *C*_peak_ and any circulating angiogenic factor.

### [^18^F]Fluciclatide is a biomarker for pazopanib

#### Per patient analysis

**Fig. 1:**
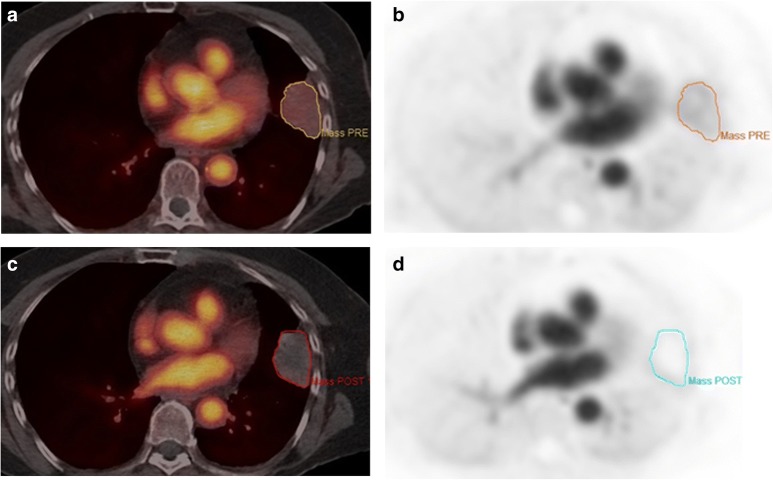
Baseline [^18^F]fluciclatide-axial fused PET/CT image (**a**) and corresponding PET image (**b**) with increased tracer uptake in left upper lobe pulmonary metastasis. Post 1 week of therapy while the lesion is still visualized on [^18^F]fluciclatide-PET/CT image (**c**), the corresponding PET image (**d**) shows a complete response to therapy

There was a median overall reduction in [^18^F]fluciclatide uptake of SUV_60,mean_ (− 18.9% + 20.1%) and SUV_60,max_ (− 14.9% + 14.4%) following the first week of pazopanib (Fig. 1). There was no association observed between either baseline SUV_60,mean_ or SUV_60,max_ and tumor response (RECIST 1.1) after 3 cycles of combination therapy (Student’s *t* test, *p* > 0.05). Furthermore, changes in SUV_60,mean_ following 1 week of pazopanib did not predict clinical response (RECIST 1.1) (ANOVA, *p* > 0.05). When considering the relationship between PET uptake parameters and PFS, a negative trend was observed between baseline SUV_60,mean_ and PFS (HR 1.75, 95%CI 0.93–3.30, *p* = 0.08). We then considered those patients whose PFS was longer than 12 months (*n* = 5); these patients had a lower SUV_60,mean_ (mean 1.65 + 0.63) compared with those with PFS less than 12 months (*N* = 9), (mean 3.21 + 1.02) (*p* = 0.01; Supplementary Fig. 3).

We further considered the relationship between baseline circulating pro-angiogenic cytokines and change in [^18^F]fluciclatide uptake. A significant positive correlation was observed between change in SUV_60,mean_ and baseline levels of angiopoietin-1 (Spearman’s rho 0.58, *p* = 0.037) and FGF (Spearman’s rho 0.56, *p* = 0.046), such that elevated levels of baseline cytokines predicted for a greater reduction in [^18^F]fluciclatide uptake.

#### Per lesional analysis

**Fig. 2:**
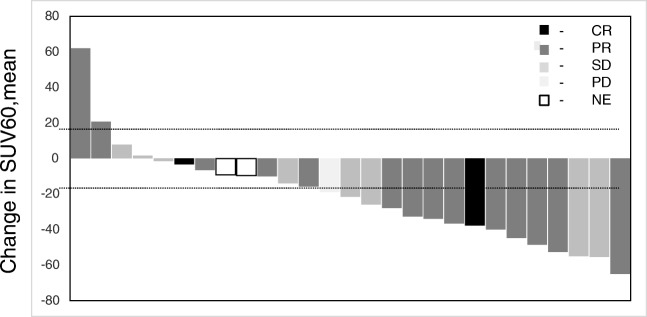
Waterfall plot illustrating the change in SUV_60,mean_ with CT response individual lesions following treatment with pazopanib. Dotted line indicates 18% variation in SUV_60,mean_ illustrating the change required to be of clinical significance. CR, complete response; PR, partial response; SD, stable disease; PD, progressive disease; NE, not evaluable

Twenty-seven lesions were detectable on PET imaging (Table 2). The average SUV_60,mean_ and mean SUV_60,max_ on the baseline scan were 2.44 (± 1.14) and 4.17 (± 1.69), respectively. When considering changes in mean SUV_60,mean_ (±SD) with CT response, the mean change in lesions undergoing CR was − 20.53 (+ 24.29), PR − 23.68 (+ 32.91), SD − 20.50 (+ 24.28), and PD − 18.9 (Fig. 2). Previous work by our group defined an objective response to [^18^F]fluciclatide statistically as a change in SUV_60,mean_ outside the 95% confidence limits, such that for any given lesion a change in SUV > 18% will be outside these limits and classified as a [^18^F]fluciclatide response [41]. Based on this, [^18^F]fluciclatide response was seen in 15 of 27 lesions (56%), with 2 lesions no longer being visible on post-treatment scans (Fig. 2). When considering response according to PET criteria, there was a greater decrease in SUV_60,mean_ in responding lesions compared with non-responding lesions; the median decrease in responding lesions was − 37.71% compared with an increase of + 4.97% in non-responding lesions (Student’s *t* test, *p* < 0.001).

**Table 2:** Lesional characteristics on [^18^F]fluciclatide imaging

Pt no.	Lesion location	SUV_60,mean_ (preRx)	SUV_60,max_ (preRx)	Lesional response	Percentage change SUV_60,ave_	Percentage change SUV_60,max_
1A	Gastrosplenic/peritoneal deposit	4.42	6.97	PR	− 33.98	− 29.65
2A	Right paracolic gutter	1.83	3.13	PR	− 6.58	9.74
2B	Mesorectal nodule	0.91	1.9	SD	7.73	− 6.32
2C	Right obturator node	2.00	2.48	PR	− 10.00	− 0.40
2D	Para-aortic node	3.13	4.44	CR	− 3.35	22.86
3A	Vaginal vault mass	4.55	8.23	PR	− 32.67	− 18.72
3B	Left obturator node	3.99	5.88	PR	− 39.92	− 31.04
3C	Left common iliac node*	3.73	5.38	CR	–	–
4A	Right obturator node	4.37	6.45	PR	− 15.79	− 14.96
5A	Anterior pelvic mass	1.26	4.62	SD	− 25.89	3.03
5B	Posterior pelvic mass	0.36	4.25	PR	61.97	3.29
6A	Peritoneal deposit	2.52	4.57	SD	− 13.92	− 9.63
6B	Subcapsular deposit	2.67	4.06	SD	− 1.50	− 5.55
6C	Para-aortic node	2.23	3.36	SD	1.57	0
8A	Right lower lobe pulmonary metastasis	1.18	1.42	CR	− 37.71	− 38.87
8B	Splenic lesion	2.78	3.52	SD	− 21.62	8.95
8C	Liver lesion	2.99	3.34	SD	− 54.92	− 39.43
9A	Left adnexal mass	1.56	3.14	NE	− 9.62	− 7.97
9B	Peritoneal lesion	1.25	1.93	NE	− 9.24	32.64
10A	Splenic lesion	3.46	4.32	PR	− 64.98	− 18.54
10B	Para-aortic node	2.87	6.99	PR	− 48.52	− 51.61
13A	Pelvic mass	3.62	5.99	PD	− 18.92	1.58
14A	Peritoneal mass	3.11	5.28	SD	− 55.47	− 20.66
103A	Vaginal mass	1.21	4.03	PR	20.66	4.96
104A	Pulmonary mass	2.39	3.38	PR	− 52.61	− 20.15
104B	Nodal mass	1.43	3.09	PR	− 44.76	3.55
104C	Left upper lobe pulmonary metastasis	1.97	2.96	PR	− 36.54	− 34.46
104D	Apical nodal mass	1.89	2.79	PR	− 27.85	− 26.52

*CR* complete response, *PR* partial response, *SD* stable disease, *NE* not evaluable

*Lesion not seen on repeat PET imaging

In patients with more than one target lesion (*N* = 8), differential lesional responses to treatment were seen in three patients on CT imaging such that they had responding lesions (CR and PR) and stable lesions on CT imaging. Poor agreement was observed between lesion response on PET imaging after 1 week of single-agent pazopanib and CT imaging response assessment after 3 cycles of therapy (kappa 0.076, *p* = 0.5). Response by PET criteria did not predict for PFS or OS.

**Fig. 3:**
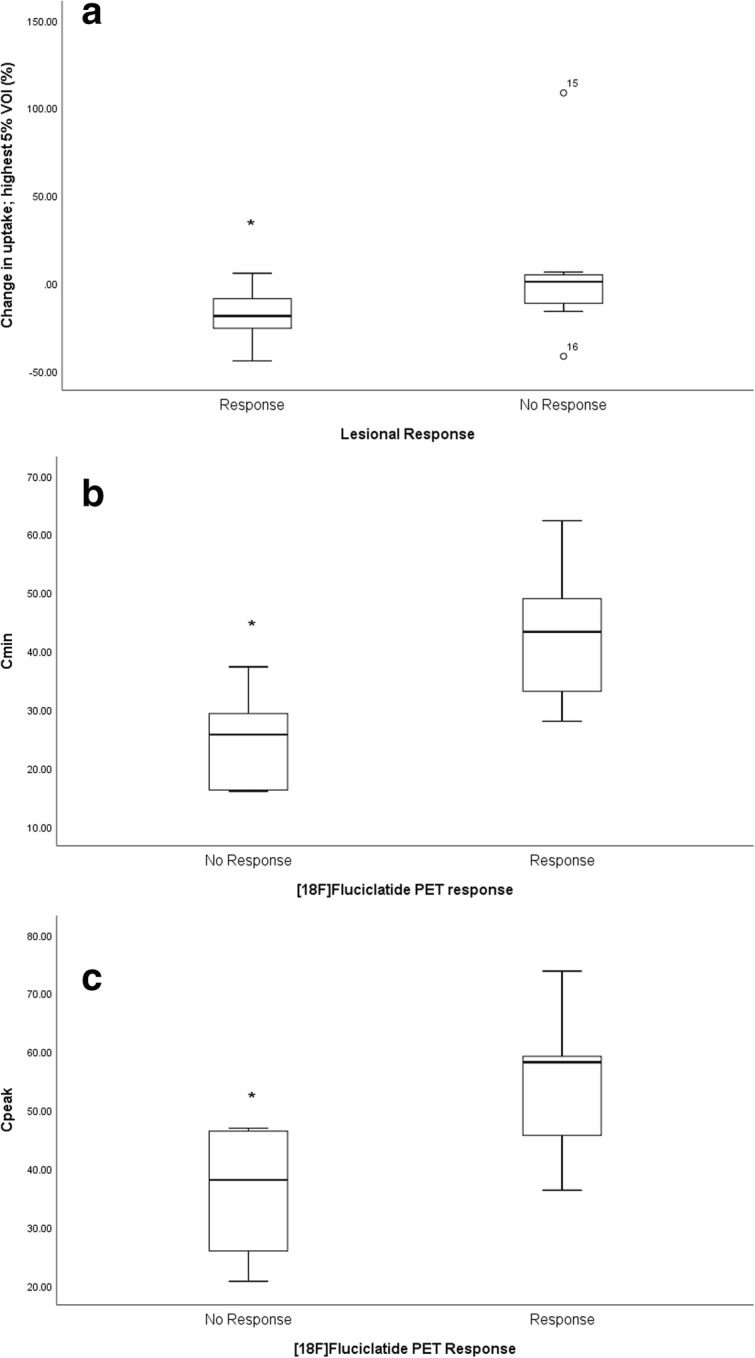
Box plot illustrating a significant relationship between lesional response and change in uptake in highest 5% voxel uptake; *p < 0.05 (**a**). Box plot illustrating a significant relationship between [^18^F]fluciclatide-PET response and *C*_min_; *p < 0.05 (**b)**. Box plot illustrating a significant relationship between [^18^F]fluciclatide-PET response and *C*_peak_; *p < 0.05 (**c**)

We explored the relationship between *C*_min_ and *C*_peak_ with PET response parameters. A significant association was observed between both *C*_min_ and *C*_peak_ and response to pazopanib as determined by [^18^F]fluciclatide-PET response (ANOVA, *p* < 0.01; Fig. 3b and c).

## Kinetic modeling illustrates significant reduction in uptake and retention parameters with pazopanib treatment

Twelve patients had blood taken for kinetic modeling. The analysis of [^18^F]fluciclatide dynamic data was undertaken using SA and graphical plots (Supplementary Table 2). SA provided estimates of both transport (*K*_1_) and retention (*K*_i_) of [^18^F]fluciclatide. Patlak plot resulted with the irreversible rate constant *K*_i_, while Logan plot resulted with the volume of distribution *V*_T_. There was a significant and profound reduction in median SA-K_1_ values from baseline (0.0024 ± 0.0019 mL/min/g), following 1 week of therapy (0.0009 ± 0.0004 mL/min/g) (Student’s *t* test, *p* = 0.006). Of interest, the percentage variation in SA-*K*_1_ was always negative, with a median reduction of 62.5% (+ 24.5). This is in keeping with a reduction in tumor perfusion following 1 week of pazopanib therapy resulting in reduced transport of [^18^F]flucliclatide to the tumor. There was a significant reduction in median Patlak derived *K*_i_ values from baseline (0.000624 ± 0.0005 mL/cm^3^/min) compared with post-treatment (0.0004 ± 0.0003 mL/cm^3^/min) (Student’s *t* test, *p* = 0.02). Similarly, there was a reduction in Logan-derived *V*_T_, 0.079 ± 0.0618 mL/cm^3^) and 0.048 ± 0.0241 mL/cm^3^) for pre- and post-treatment scans, respectively (Student’s *t* test, *p* = 0.013). All but one patient had a reduction in *V*_T_, median reduction – 59.61% (± 29.94). Despite the marked and significant changes in the PET uptake and retention parameters, no association was observed between changes in any PET uptake parameter and response to 3 cycles of combination therapy (RECIST 1.1). Baseline SUV_60, mean_ and change in *K*_i_ and *V*_T_ were significantly related (Spearman rho correlation coefficient − 0.62, *p* = 0.03, for both), and a significant relationship was observed between baseline *V*_T_ and baseline SUV_60, mean_ (Spearman rho correlation 0.89, *p* < 0.001).

### [^18^F]Fluciclatide uptake at disease progression

**Fig. 4:**
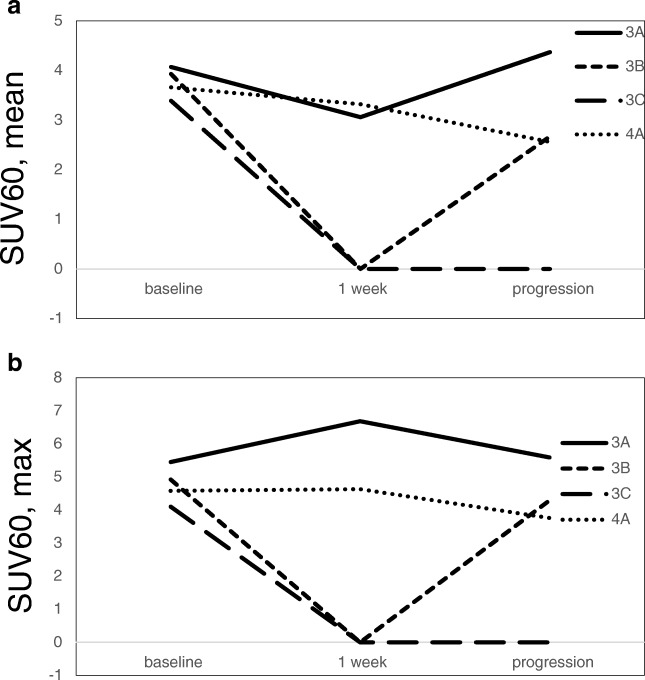
Line graph illustrating changes in SUV_60,mean_ in four lesions at baseline, after 1 week of pazopanib therapy and at disease progression (**a**). Line graph illustrating changes in SUV_60,max_ in four lesions at baseline, after 1 week of pazopanib therapy and at disease progression (**b)**

Only two patients (003 and 004) agreed and underwent PET imaging on disease progression. Due to the small sample size, no formal statistical analysis was undertaken. Both patients had serous papillary, stage IV ovarian cancer at the time of enrolment. Subject 003 had had two previous lines of therapy prior to study enrolment, while 004 had only had one. Subject 003 completed combination therapy and remained in the study for 11.3 months, while 004 relapsed outside of the PET field of view within 5.2 months of commencing combination treatment. Subject 003 had three lesions detected on baseline imaging: a vaginal vault mass, left obturator node, and a left common iliac node. CR was observed in the left common iliac node following 3 cycles of combination therapy, while the other two lesions underwent PR according to CT criteria, and as reflected by a reduction in [^18^F]fluciclatide uptake (Fig. 4a and b). Subject 004 had only 1 lesion (the right obturator node) that underwent PR on CT imaging, although no reduction in [^18^F]fluciclatide uptake was observed after 1 week of pazopanib. On disease progression, a sharp increase in tracer uptake was observed in the vaginal vault mass and left obturator node (subject 003), suggesting an increase in angiogenesis associated with pazopanib combination therapy. Subject 004 progressed outside of the PET field of view. At this time, CT imaging of the right obturator node showed SD. This was in keeping with [^18^F]fluciclatide imaging which did not show any increased tracer uptake.

## Discussion

We have shown that in patients with platinum-resistant ovarian cancer, the combination of pazopanib and weekly paclitaxel followed by maintenance pazopanib is effective and tolerable. Importantly, we have also shown that [^18^F]fluciclatide-PET is a biomarker of the anti-angiogenic effect of pazopanib such that high baseline uptake on PET imaging was predictive of a PFS of less than 12 months in patients with platinum-resistant/refractory ovarian cancer.

Two previous randomized controlled studies have investigated the role of combination of pazopanib and paclitaxel in the management patients with recurrent ovarian cancer [14, 17]. The study by Richardson and colleagues reported no differences in clinical outcome in patients receiving paclitaxel and pazopanib compared with paclitaxel and placebo. The study illustrated an overall response rate (CR + PR) of 26% and an OS of 20.7 months with combination therapy. In contrast, the MITO 11 study reported a definite improvement in PFS with combination therapy, with an overall response rate of 64%. The results from the MITO 11 study are in line with the AURELIA study which report an improvement in PFS with the addition of bevacizumab to chemotherapy in platinum-resistant disease [9]. The differences in clinical outcome between the MITO II and Richardson studies can be primarily attributed to differences in the population studied, whereby MITO 11 only enrolled patients with platinum-resistant or refractory disease while over half the study population in the Richardson study had platinum-sensitive disease. Furthermore, in the latter study, 20% of patients had had prior bevacizumab therapy which was likely to impact the outcome of future challenge with anti-angiogenic therapy. In line with the MITO 11 and AURELIA studies, we report PFS of 7.97 months and an equivalent response rate. However, we report a longer OS in keeping with the Richardson study, suggesting an additive survival benefit in continuing maintenance pazopanib. The therapeutic activity of this approach is suggested by the 64% of patients rendered “platinum sensitive” in this study such that a significant number had a favorable response to platinum re-challenge. Maintenance pazopanib and the triple angiokinase inhibitor (nintedanib (BIBF 1120)) have shown clinical benefit in the platinum-sensitive setting; recently, the promiscuous tyrosine kinase inhibitor sorafenib has been shown to prolong PFS when used as maintenance therapy in the platinum-resistant setting [16, 42, 43]. However, this is the first report of a pan-VEGF inhibitor approach as maintenance therapy showing activity in the platinum-resistant setting and does warrant further investigation in the platinum-resistant/refractory population. The adverse events experienced by patients in this study are consistent with those published in the literature. Of note, adverse events did not result in the cessation of pazopanib in the maintenance setting. Hence, we can conclude that this proof of concept study shows efficacy of combination therapy followed by maintenance suppression of angiogenesis in platinum-resistant/refractory disease that should be pursued further.

While anti-angiogenic therapy has been shown to improve PFS in patients with both platinum-sensitive and platinum-resistant ovarian cancer, there are few validated biomarkers of response to these treatments, not only in ovarian cancer but in any cancer type [9, 10, 12, 14]. Preclinical studies indicate that anti-angiogenic therapies cause vascular normalization, reduction in vessel density, and increased vessel maturity, resulting in improved delivery of cytotoxic agents, the central hypothesis in combining anti-angiogenics with chemotherapy [44]. Imaging the time point of vascular normalization would allow optimal timing of cytotoxic delivery, improving clinical outcome [45]. The most widely used imaging technique for visualization of vascularization is dynamic contrast-enhanced (DCE)-MRI. DCE-MRI enables the assessment of vascular flow and capillary permeability through the measure *K*^trans^ (volume transfer constant). *K*^trans^ has been shown to be significantly reduced following the administration of multiple anti-angiogenic compounds including pazopanib, where the *C*_max_ of pazopanib correlated with changes in *K*^trans^ [46, 47]. Several studies have suggested that changes in *K*^trans^ may be an early pharmacodynamic biomarker of clinical response to anti-angiogenics [48–50]. However, *K*^trans^ denotes a context-dependent (including degree of tumor perfusion and MRI acquisition setup/equipment) complex combination of tissue blood flow and permeability weighted to varying extents. In tumors where the variable largely represents perfusion due to limited contrast delivery however, *K*^trans^ can transiently increase with anti-angiogenic therapy as a result of vascular normalization and changes in local vasodilatory factors, followed by a reduction associated with decreases in neovasculature and endothelial cell apoptosis (vascular pruning); hence, timing of scanning, type of contrast, and sequence can make assessment of changes in DCE-MRI parameters difficult to interpret [51, 52]. Moreover, *K*^trans^ remains an indirect measure of angiogenesis, as it is a measure of vascular permeability influenced by blood flow, capillary surface area, and physiological factors [53], and the lack of standardization of imaging acquisition and data analysis with DCE-MRI also remains a clinical limitation. Radionuclide imaging using [^18^F]fluciclatide allows the specific molecular imaging of α_v_β_3/5_-integrins on the neovasculature, indicative of MVD [54]. The central hypothesis of our study is that the use of the anti-angiogenic agent pazopanib results in a reduction in MVD and vessels expressing α_v_β_3/5_-integrins as indicated by a reduction in [^18^F]fluciclatide uptake. This hypothesis is supported by the preclinical work by Battle and colleagues who illustrated a reduction in [^18^F]fluciclatide uptake and MVD in tumors treated with sunitinib [28]. Of interest, we observed that patients with PFS > 12 months had lower baseline tumor [^18^F]fluciclatide uptake than those with PFS < 12 months, even though one would intuitively expect tumors with high [^18^F]fluciclatide uptake, thus greater expression of α_v_β_3/5_-integrins, to respond better to antiangiogenic therapy. Of note, higher MVD is associated with worse prognosis and this may explain the inverse association of PFS with baseline fluciclatide uptake, a finding supported by the work of Rubatt and colleagues, which illustrate that increased MVD is associated with worse PFS in patients with ovarian cancer [6]. We found no relationship between changes in [^18^F]fluciclatide uptake with response according to RECIST 1.1, a finding alluded to by Beer and colleagues who reported no relationship between tumor size and tracer uptake [55]. There are a number of possible explanations for this. Firstly, PET response was assessed after 1 week of single-agent pazopanib while RECIST 1.1 response was performed after 3 months of combination therapy; therefore, the two imaging modalities are assessing potentially differing therapeutic processes. It is well established that single-agent anti-angiogenics result in predominantly cytostatic response while the addition of chemotherapy enables reduction in tumor size which may account for the lack of association observed. Patients in this study underwent dynamic imaging in 1 bed position. PET response is therefore based only on those target lesions within the field of view, while RECIST 1.1 assessment was based on whole-body imaging. Single-bed, dynamic scans were conducted in order to understand the effect of the drug tracer kinetics, consequently to confirm the most practical time for imaging when [^18^F]fluciclatide is used as an imaging test in monitoring the effect of antiangiogenic therapy. We anticipate that future studies employing [^18^F]fluciclatide will build on our work to implement a more standard static, multi-bed whole-/half-body imaging protocol in a larger study population [41]. Finally, preclinical work suggests that normalization of vessels in response to anti-angiogenics occurs within hours and lasts 7–10 days, and the optimal timing of imaging this process remains to be established, and it is possible that performing the second PET scan at 7 days may have missed the neovascularization window [45].

In terms of PKs of pazopanib, a number of studies suggest that a *C*_min_ of > 20 mg/L is associated with tumor response and survival, suggesting the role for PK-guided dosing [40, 56]. On lesional analysis, we observed a significant correlation between plasma levels of pazopanib and response according to PET criteria such that a higher concentration of pazopanib correlated with PET response. These findings are consistent with the anti-angiogenic effects of the drug, such that a higher concentration will have a greater impact on pharmacodynamic (PD) endpoint illustrating a PK-PD relationship, as supported by Yau and colleagues who report a significant correlation between *C*_max_ of pazopanib and *K*^trans^ following treatment in patients with hepatocellular cancer [46].

Again, consistent with the PD effects of pazopanib are the results of the dynamic PET modeling. We performed kinetic modeling using a 2-compartmental model approach, and the values of PET parameters obtained were in keeping with previous work [57]. In general baseline, *V*_T_ values were low (< 0.1 mL/cm^3^) which perhaps suggest physiologically low receptor density of α_v_β_3/5_ in ovarian tumor lesions. We observed a significant reduction in *K*_1_ and *K*_i_ following 1 week of pazopanib, illustrating a reduction in delivery of the PET ligand to the tumor mass and reduced tissue retention. Moreover, we also observed a significant relationship between baseline SUV_60,mean_ and changes in *K*_i_ such that higher SUV_60,mean_ was associated with a larger reduction in *K*_i_ indicating a greater reduction in ligand retention following pazopanib therapy.

While only in a small number of patients, we also show an increase in [^18^F]fluciclatide uptake in those lesions that progressed on CT imaging following pazopanib therapy. Resistance to VEGF inhibitors is inevitable, the mechanism of which is postulated to be the overexpression and stabilization of hypoxia-inducible factor (HIF)-1α that directly regulates VEGF expression, and our results clearly illustrate this failure of functional blockade [58]. Taken together, the [^18^F]fluciclatide-PET data suggest that the PET parameters obtained are consistent with the PD endpoint of pazopanib.

Numerous studies have considered the predictive relationship between circulating levels of pro-angiogenic cytokines and response to anti-angiogenics including pazopanib [59–61]. The prognostic value of VEGF-A is not clear, and more recently SNPs in VEGF-A and VEGFR1 have shown promise in randomized trials [15, 62, 63]. We found that only baseline levels of circulating levels of angiopoietin-1 correlated with a reduction in tumor size in keeping with published literature; however, no relationship was observed with PKs [64]. It is unlikely that any one cytokine will be able to predict response to anti-angiogenic agents, and given the small sample size, any findings should be interpreted with caution. However, of particular interest was the association between baseline circulating levels angiopoietin-1/FGF and changes in SUV_60,mean_, such that increasing levels of cytokines at baseline correlated with a greater change in this PET parameter. Angiopoietin-1 and FGF play a central role in vascular development and angiogenesis, and this finding may be reflective of a greater change in MVD in response to pazopanib. This finding would be strengthened by histologic corroboration, a key limitation of our study, and future work should include tumor biopsy at the start of therapy and after angiogenic therapy to establish true surrogacy between PET uptake parameters and MVD.

In conclusion, we report that the combination of pazopanib and paclitaxel followed by maintenance pazopanib is an effective, tolerable regimen in the management of platinum-resistant/refractory ovarian cancer that should be taken forward in larger studies. Furthermore, for the first time in human subjects, we have shown that [^18^F]fluciclatide-PET is a PD marker of pazopanib response.

## Electronic supplementary material

ESM 1 (DOCX 13 kb)

ESM 2 Figure 1. Change in serum CA125 according to GCIG CA125 criteria according to number of weeks on treatment. Each line indicates an individual subject as indicated by the allocated subject number. Responders (*n* = 11) and non-responders (subject number 13) (*n* = 1) are indicated. Figure 2A. Kaplan-Meier analysis of progression free survival of entire study population (*n* = 14). Figure 2B. Kaplan-Meier analysis with log rank test of progression free survival indicating significant difference in survival according to response using RECIST 1.1. CR – complete response, PR – partial response, SD – stable disease, PD – progressive disease. Figure 2C. Kaplan-Meier analysis with log rank test of overall survival indicating significant difference in survival according to response using RECIST 1.1. CR – complete response, PR – partial response, SD – stable disease, PD – progressive disease. Figure 3. Box plot indicating significant difference in progression free survival (PFS) in relation to baseline SUV60,mean such that patients having a PFS < 12 months have a higher baseline SUV60,mean whilst those with a PFS > 12 months have a lower baseline SUV60,mean. ANOVA. * p0 < 0.05. (PPTX 93 kb)
