# Supplementary material for: [18F]Fluciclatide PET as a biomarker of response to combination therapy of pazopanib and paclitaxel in platinum-resistant/refractory ovarian cancer
Source: Eur J Nucl Med Mol Imaging. 2019 Nov 21;47(5):1239–51. doi: 10.1007/s00259-019-04532-z (PMC7101300; doi:10.1007/s00259-019-04532-z)
Supplement: Supplementary file 1 — (DOCX 13 kb) [file 259_2019_4532_MOESM1_ESM.docx]

| **Proangiogenic factor** | | **Median baseline value (pg/mL)** | **Median fold change** |
| --- | --- | --- | --- |
| PIGF | | 3.0 | 0.58* |
| TIE-2 | | 24825.6 | -0.044 |
| VEGF | | 114.9 | -0.44 |
| VEGF-C | | 333.1 | -0.031 |
| Endothelin-1 | | 178.7 | 0.0099 |
| Endostatin | | 71908.0 | -0.094 |
| VEGF-D | | 1583.3 | 0.037 |
| HGF | | 1859.9 | -0.009 |
| Angiopoietin-1 | | 45077.9 | -0.11 |
| VEGFR3 | | 2520.1 | -0.11 |
| FGF | | 108.6 | -0.52 |
| GMCSF | | 166.0 |  |
| VEGFR1 | | 3212.1 | -0.18 |
| VEGFR2 | | 22572.7 | -0.12 |
| *significant change compared to baseline p<0.05  **Supplementary table 1** Levels of circulating cytokines at baseline and following 1 week of pazopanib therapy   \| **Patient Number** \| **K_1_ (mL/cm^3^/min)** \| **V_T_ (mL/cm^3^)** \| **K_i_ (mL/cm^3^/min)** \| \| --- \| --- \| --- \| --- \| \| 001 \| 0.0025 \| 0.18 \| 0.0015 \| \| 003 \| 0.0031 \| 0.21 \| 0.0017 \| \| 004 \| 0.0032 \| 0.11 \| 0.00096 \| \| 005 \| 0.0076 \| 0.02 \| 0.00017 \| \| 006 \| 0.0016 \| 0.09 \| 0.00072 \| \| 008 \| 0.0038 \| 0.02 \| 0.00018 \| \| 009 \| - \| - \| - \| \| 010 \| 0.0013 \| 0.07 \| 0.00062 \| \| 013 \| 0.0023 \| 0.05 \| 0.00019 \| \| 014 \| 0.0018 \| 0.05 \| 0.00042 \| \| 103 \| 0.0033 \| 0.09 \| 0.00072 \| \| 104 \| 0.0009 \| 0.10 \| 0.00085 \|   **Supplementary table 2**. Lesional characteristics on [^18^F]fluciclatide imaging using Spectral and Graphical analysis | | | |
