# Supplementary material for: [18F]Fluciclatide PET as a biomarker of response to combination therapy of pazopanib and paclitaxel in platinum-resistant/refractory ovarian cancer
Source: Eur J Nucl Med Mol Imaging. 2019 Nov 21;47(5):1239–51. doi: 10.1007/s00259-019-04532-z (PMC7101300; doi:10.1007/s00259-019-04532-z)
Supplement: Supplementary file 2 — Figure 1. Change in serum CA125 according to GCIG CA125 criteria according to number of weeks on treatment. Each line indicates an individual subject as indicated by the allocated subject number. Responders (n = 11) and non-responders (subject number 13) (n = 1) are indicated. Figure 2A. Kaplan-Meier analysis of progression free survival of entire study population (n = 14). Figure 2B. Kaplan-Meier analysis with log rank test of progression free survival indicating significant difference in survival according to response using RECIST 1.1. CR – complete response, PR – partial response, SD – stable disease, PD – progressive disease. Figure 2C. Kaplan-Meier analysis with log rank test of overall survival indicating significant difference in survival according to response using RECIST 1.1. CR – complete response, PR – partial response, SD – stable disease, PD – progressive disease. Figure 3. Box plot indicating significant difference in progression free survival (PFS) in relation to baseline SUV60,mean such that patients having a PFS < 12 months have a higher baseline SUV60,mean whilst those with a PFS > 12 months have a lower baseline SUV60,mean. ANOVA. * p0 < 0.05. (PPTX 93 kb) [file 259_2019_4532_MOESM2_ESM.pptx]

## Slide 1
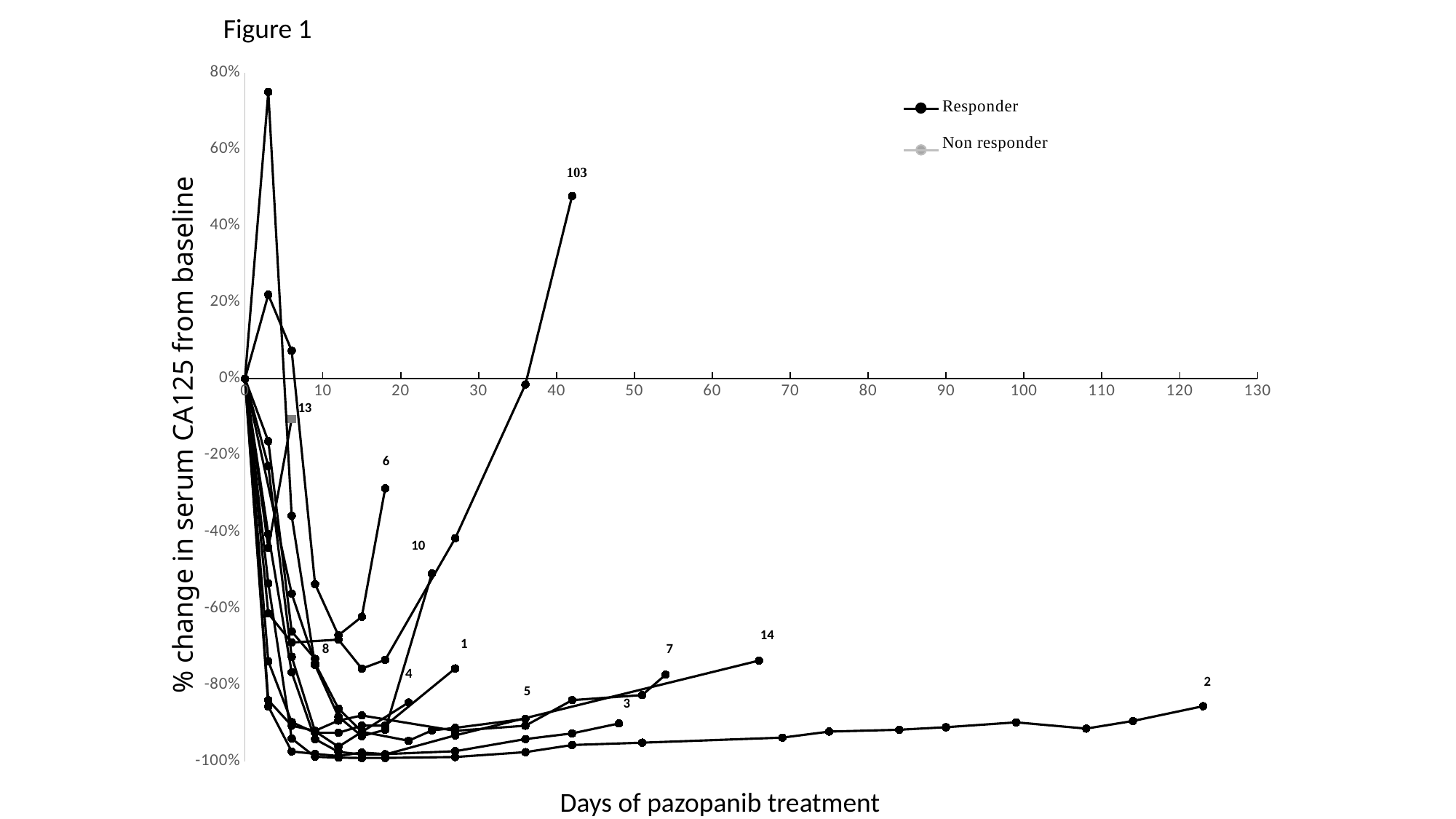

Figure 1
### Chart
| Category | 1 | 2 | 3 | 4 | 5 | 6 | 7 | 8 | 10 | 13 | 14 | 103 |
|---|---|---|---|---|---|---|---|---|---|---|---|---|% change in serum CA125 from baseline
Days of pazopanib treatment

## Slide 2
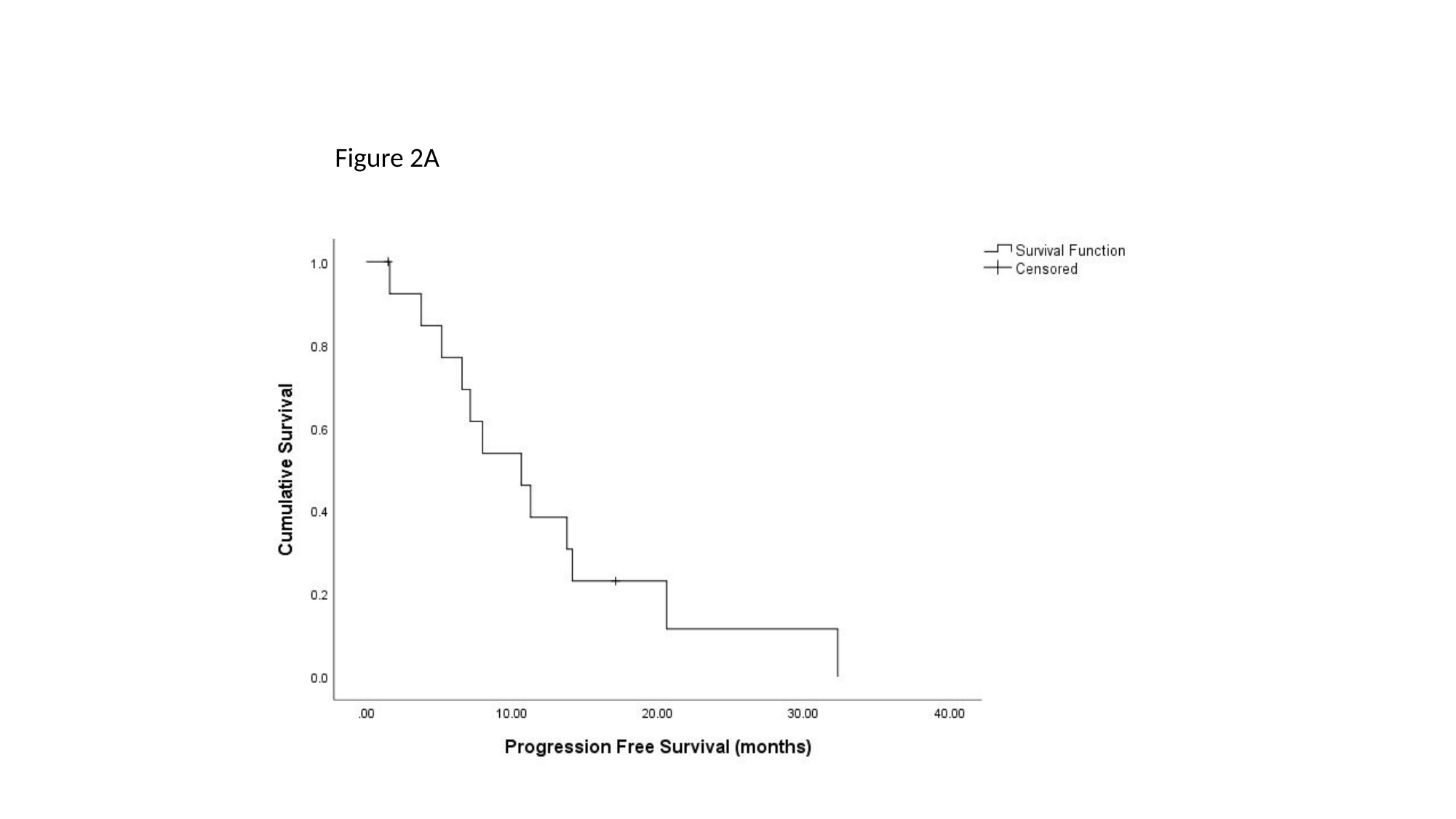

Figure 2A

## Slide 3
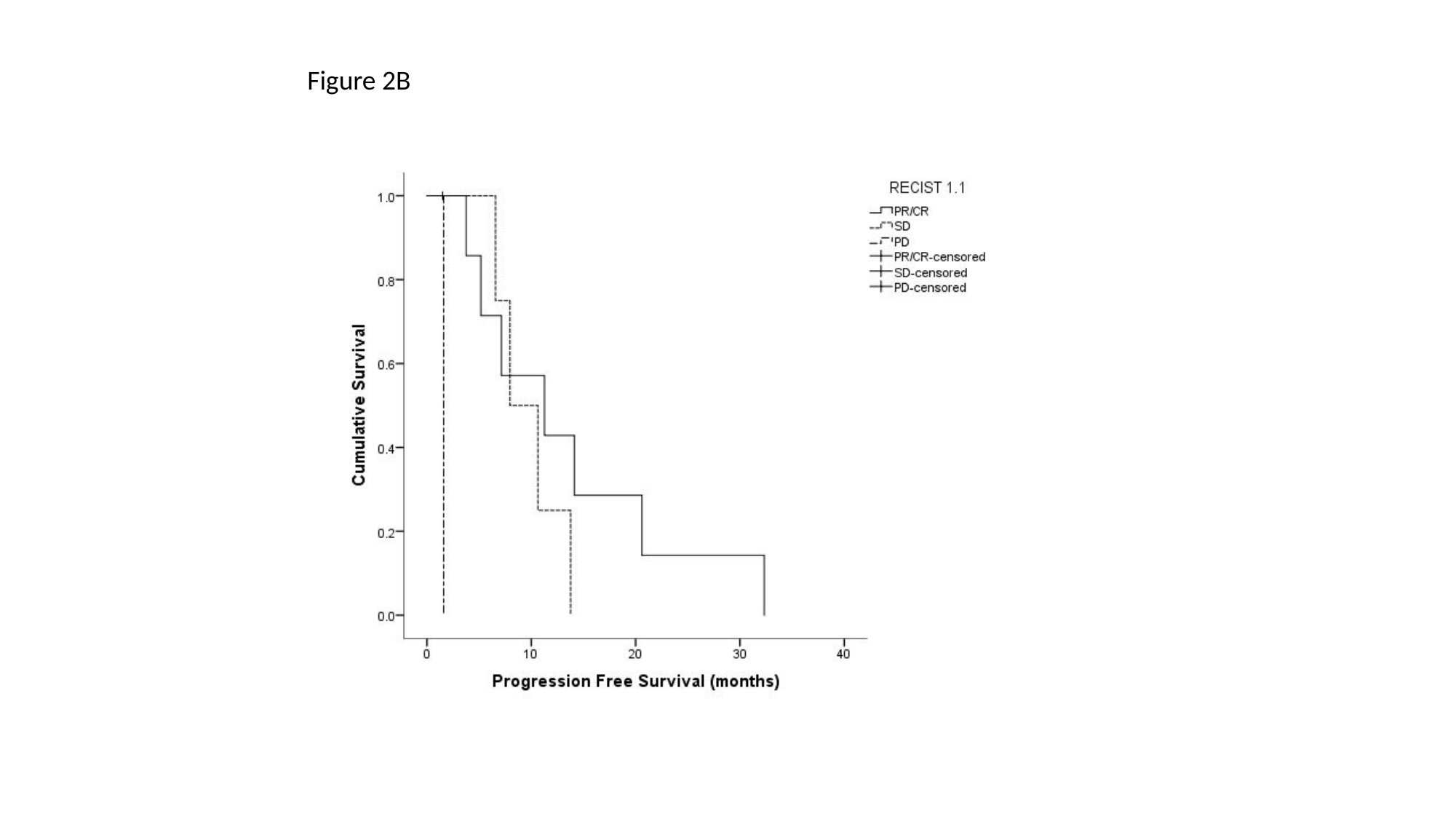

Figure 2B

## Slide 4
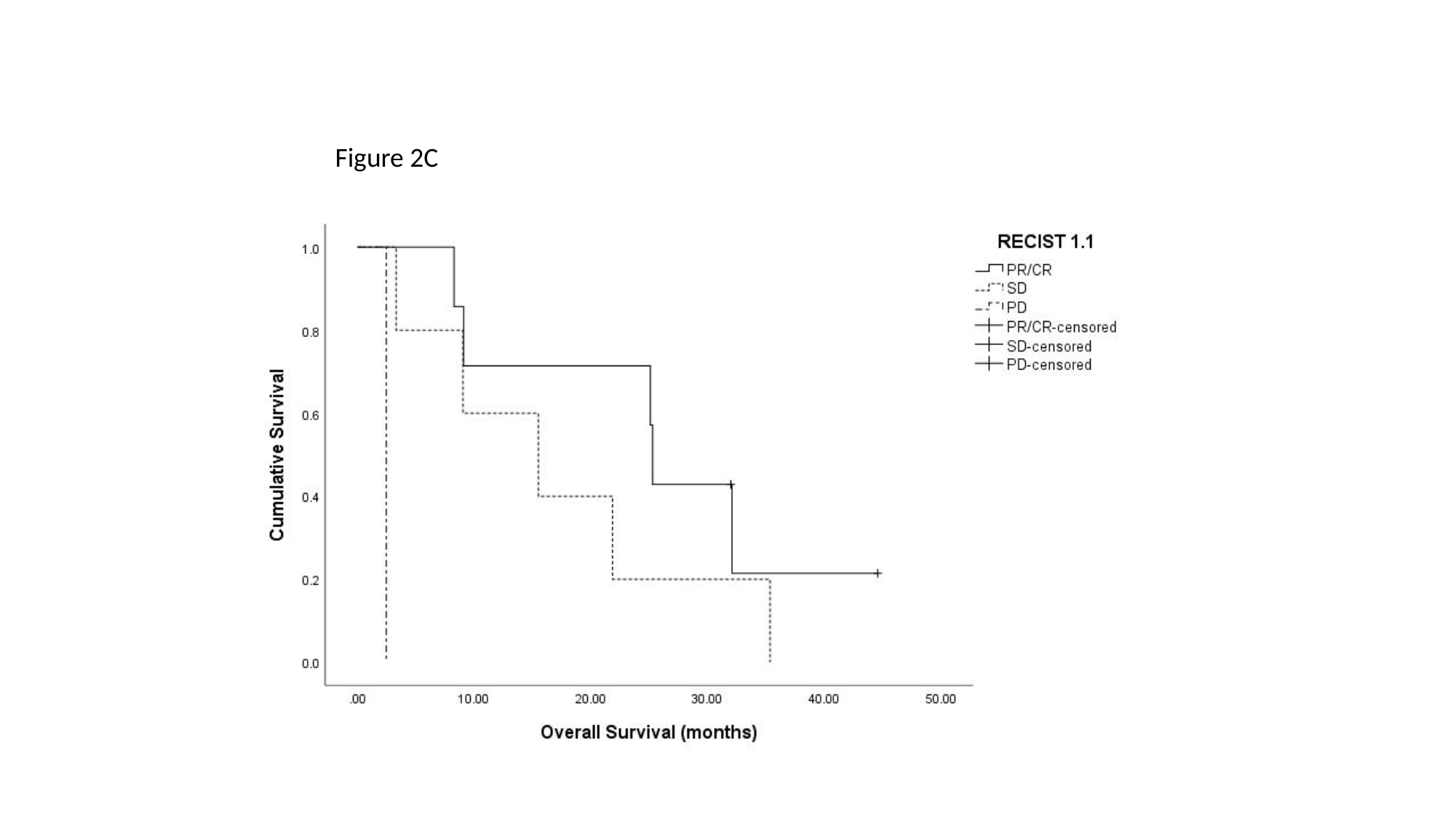

Figure 2C

## Slide 5
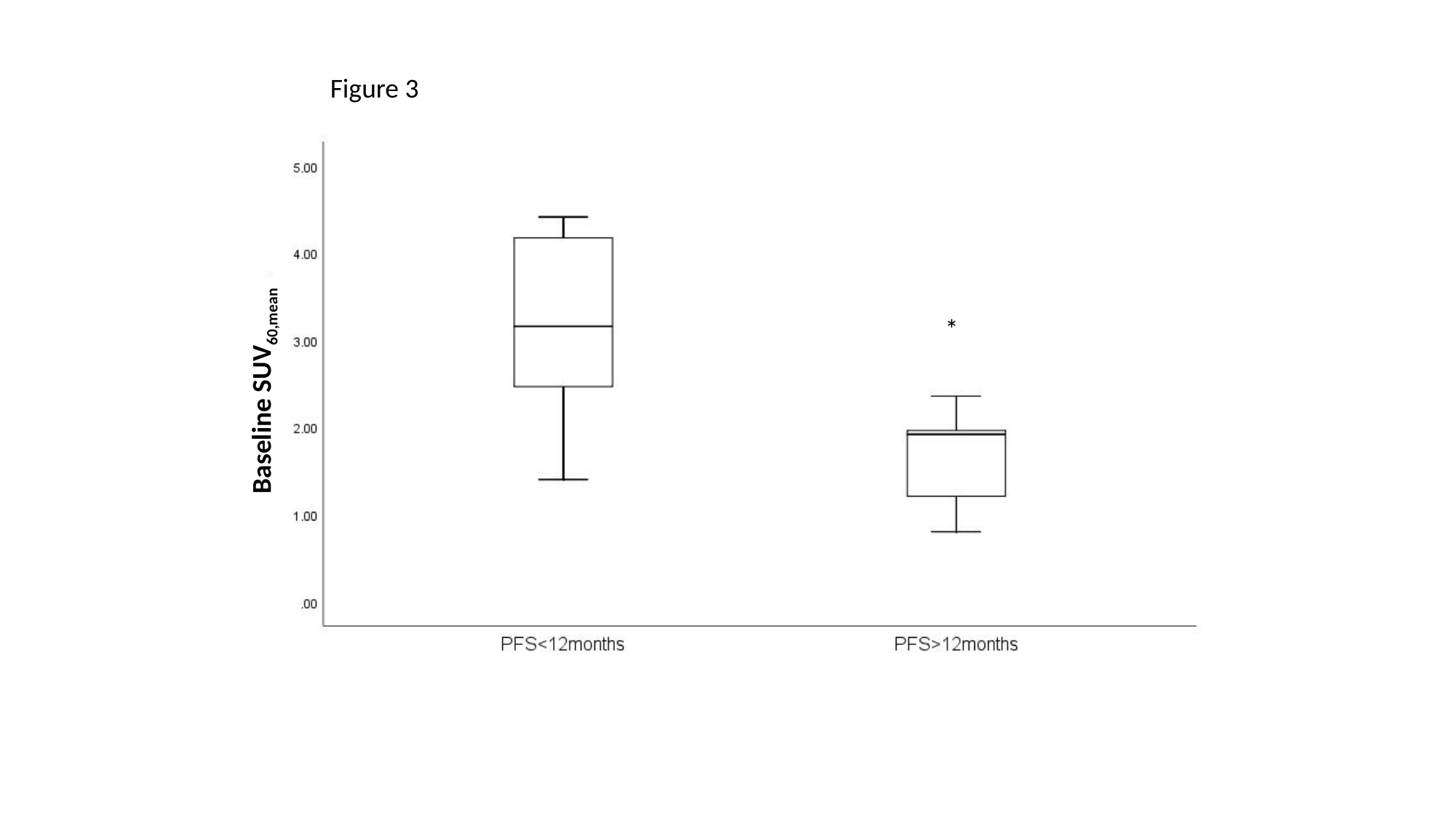

Figure 3
*
Baseline SUV60,mean
